# “It opened a new door for me”: A qualitative study of forcibly displaced parents’ experiences of an attachment-based parenting program

**DOI:** 10.1177/13591045231202875

**Published:** 2023-09-20

**Authors:** Anna Kristen, Marlene M Moretti, Fatumo Osman

**Affiliations:** 1 Department of Psycholog 1763 y, Simon Fraser University, Canada; 2 School of Health and Welfare, 3317 Dalarna University, Sweden

**Keywords:** Parent-teen relationships, attachment, parenting, forced displacement, adolescents

## Abstract

The aim of the study was to explore forcibly displaced parents’ experiences of how an online attachment-based parenting program (*eConnect*) impacted their relationships with their teens. Data was collected from four focus group discussions with 28 parents who participated in the *eConnect* program. Data was analyzed using network thematic analysis. A global theme emerged from the analysis: *Strengthened Parent-Teen Relationships*. Four underpinning organizing themes described the process through which the parent-teen relationship was strengthened: *Knowledge Served as the Foundation for Change, Increased Parental Self-Efficacy, Improved Emotional Attunement Facilitates Dyadic Affect Regulation, and Shifted Power Dynamics and Emerging Mutual Parent-Teen Partnership*. Findings suggest that *eConnect* is promising intervention for strengthening parent-teen relationships and supporting forcibly displaced families.

## Introduction

Secure parent-child attachment relationships are one of the most important determinants of youth development and well-being (Bosmans & Borelli, 2022). Extensive research has shown that sensitive and responsive parenting is foundational in developing secure attachment relationships (Bosmans et al., 2020); however, parents who have experienced forced displacement report challenges parenting due to the migration experiences that may compromise their capacity for sensitive and responsive parenting. Traumatic pre-displacement experiences and acculturative stressors in their host country including unemployment, limited social support, and language challenges, create significant parenting challenging for forcibly displaced parents (Betancourt et al., 2015; Moinolmolki et al., 2020).

Forcibly displaced parents must also navigate the parenting role in a new country, often grappling with drastically different parental expectations, values, and norms (Osman et al., 2016). This is especially the case for forcibly displaced families from collectivistic hierarchical family systems that place emphasis on familial interdependence, obedience, and an authoritarian parenting style when migrating to westernized contexts that promote individualistic family systems. Such disparities in parenting values, expectations, and practices can give rise to parental feelings of inefficacy, helplessness, and loss of authority in the parenting role (Bergnehr et al., 2018). Moreover, studies have emphasized parenting challenges related to acculturative dissonance (Betancourt et al., 2015; Osman et al., 2021) in which parents and youth acculturate at differing rates, typically with youth acculturating more quickly. Conflict often emerges as a result of clashes between youth desires for cultural integration and autonomy and parental desires to uphold cultural values and practices. Consequently, communication breakdowns are common, and parents often report feeling disconnected from their children (Eltanamly et al., 2021).

Emerging research shows that parenting interventions that focus on improving the parent-child relationship may be a particularly promising approach for supporting forcibly displaced families (Hamari et al., 2022). While some parenting programs that are culturally and contextually relevant to families exist, they are exceptionally scarce, despite the historically high rates of forcibly displaced people worldwide (UNCHR, 2022). In addition to the limited availability of these interventions, forcibly displaced families face disproportionate barriers to accessing mental health services (e.g., geographical isolation; language barriers). The COVID-19 pandemic brought to the forefront the potential that virtually delivered programs may be a promising avenue to diminish service access disparity for low socio-economic and minority populations (Siegel et al., 2021). Yet, limited research has explored online delivered parenting programs as a potential avenue for service delivery for forcibly displaced populations.

### The program

The *Connect* program is an attachment-based and trauma-informed intervention designed for parents with adolescents experiencing significant social-emotional problems (Moretti & Obsuth, 2009). The program is delivered by two facilitators in 10 weekly, 90-min sessions. Each session, parents are introduced to an attachment principle related to attachment, adolescent development, and parenting. During sessions, parents are encouraged to “step back” from their own emotional experience and reactions to teen behaviour and to “step into” the mind of their teenagers. The program uses role plays, reflective exercises, and emotion-focused learning to promote parental reflective functioning, and to strengthen parenting sensitivity, dyadic affect regulation, and parent-teen partnership. The program can be delivered in-person or online (*eConnect*; Bao & Moretti, 2023). The online version of the program retains all components of the in-person version of the program including real-time interactive group processes.

### The current study

Our previous studies delivering a culturally tailored version of the *Connect* parenting program for Somali-born parents living in Sweden indicated that the program was effective for improving both parental and youth mental health problems, and for improving parental efficacy and satisfaction, and these treatment gains were maintained at a 3-year follow-up (Osman et al., 2017, 2021). In a recent qualitative study, we examined Somali-born parents’ experiences of the *Connect* program, and the results showed that parents gained confidence in the parenting role and became more aware of their child’s socio-emotional needs (Osman et al., 2019). While these studies provide evidence for the *Connect* program in supporting foribly displaced parents, there is limited research exploring parent experiences of the online version of the program among forcibly displaced parents. Additionally, given the unique challenges faced by forcibly displaced parents and the parenting challenges associated with the COVID-19 pandemic, it is important to explore the impact of the *eConnect* program upon parent-child relationships. Thus, the purpose of this study is to explore forcibly displaced parents’ experiences of the impact of the *eConnect* program upon their relationships with their teenagers.

## Methods

The study is a part of a larger international research program evaluating the online version of the *Connect* parenting program (*eConnect*; Bao & Moretti, 2023).

### Setting and recruitment

Parents were recruited from municipality-based activities (e.g., languages classes) in a small city in Sweden from May until August 2021, during the COVID-19 pandemic. Four *eConnect* groups were completed between June and December 2021: two Somali-speaking groups (*n* = 5, *n* = 8), one Dari-speaking group (*n* = 10), and one Arabic-speaking group (*n* = 5). All parents were invited to participate in post-program focus group discussions (FDGs), and all parents completed written informed consent to participate (*n* = 28). Ethical approval was received from the Swedish Ethical Review Authority (Dnr. 2020-06737), and the Simon Fraser University Research Ethics Board (#2011s0284 and #20200401).

### Participants

The study included forcibly displaced parents (*n* = 28; 50% mothers) of adolescents aged 8–18 years old. Parents reported their country of origins to be Afghanistan (43.5%), Somalia (46.4%), and Syria (17.9%). Parents reported having between one and 11 children (*M* = 5.15, *SD* = 2.33). Of the 25 parents that reported their employment status, the majority were unemployed at the time of starting the program (84%). Parents had held immigration status for between 2 years to 32 years (*M* = 8.52, *SD* = 8.45). All parents attended between 7 and 10 (*M* = 8.86, *SD* = .97) sessions of the *eConnect* program.

### Data collection

Four separate online FGDs were conducted post-program. The focus groups were led by the last author who is fluent in Somali, Swedish, and Arabic. A professional interpreter joined the Dari focus group. Two focus groups were held online and two were held in-person at the municipality, and the last author joined via Microsoft Teams. The two in-person focus groups were held as a part of the final celebration organized for the parents after the pandemic restrictions were lifted. We used a modified version of the interview guide (see appendix) used in a previous study with Somali-born parents (Osman et al., 2019). Semi-structured interview questions were posed to the group, and follow-up questions were focused on clarifying meaning and details. Parents were asked about their experiences of the program (e.g., positive or negative aspects of the program, most valuable components of the program and how they used what they had learned in their family). The FGDs were approximately 60 minutes each, and were recorded via Microsoft Teams.

### Data analysis

The data was professionally translated and transcribed verbatim. The data was analyzed using network thematic analysis as outlined by Attride-Stirling (2001). Thematic network analysis aims to generate themes at different levels of analysis (basic, organizing, and global themes) and provide a process for data interpretation. The thematic network is developed from basic themes which are “simple premises characteristic of the data” (Attride-Stirling, 2001, p. 389). Organizing themes are constructed to capture the essence of basic themes and taken together form the global theme which encompasses the overarching message in the data. The thematic network illustrates the interconnected nature of relationships between and within the levels of analysis.

The data was explored within the context of study aims (i.e., an inductive-deductive approach). The analysis was carried out by the first and last author. To assess methodological integrity, the authors were guided by Lincoln and Guba’s (1986) four criteria to establish trustworthiness in qualitative research: credibility, transferability, dependability, and confirmability.

The authors began with numerous readings of the dataset to facilitate data familiarization. This was followed by a line-by-line coding of data relevant to the research aims. This stage of analysis formed the basic themes. The authors completed this first stage independently to support inter-rater reliability and all four criterion of trustworthiness. Following this stage, the authors compared basic themes and resolved discrepancies via discussion until consensus was achieved. Only minor discrepancies were found (e.g., using different synonymous terms). The authors then explored patterns of basic themes within and among transcriptions to generate organizing themes. At this stage, to support credibility, the last author re-listened to the audio recorded FDGs to ensure that the themes accurately captured parents’ narratives.

The next stage of analysis involved the construction of the thematic network. In this final stage, authors described the themes and the relationship among themes using supporting excerpts from the transcripts. The analytic process was iterative and recursive, requiring the authors to move back and forth between analytic stages. The analytic process was also collaborative and reflexive, involving regular discussions between authors, peer-debriefing, and reflexive notetaking, supporting credibility, dependability, and confirmability. Although the FDGs were held separately with parents from Somalia, Syria, and Afghanistan, the themes that emerged were consistent across all groups.

## Results

**Figure 1.**
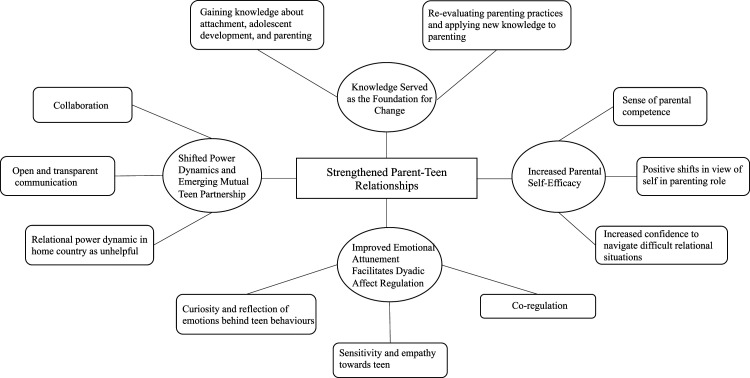
Thematic network analysis of the impact of the *Connect* program on parents and parent-teen relationships.

One global theme emerged in our analyses: *Strengthened Parent-Teen Relationships*. This overarching theme captures parents’ perceptions that the *eConnect* program strengthened their relationships with their teenagers. Four underpinning, organizing themes described the meaningful changes parents experienced in their relationships with their teenagers: *Knowledge Served as the Foundation for Change, Increased Parental Self-Efficacy, Improved Emotional Attunement Facilitates Dyadic Affect Regulation, and Shifted Power Dynamics and Emerging Mutual Parent-Teen Partnership*. The organizing themes describes a non-linear process through which the parent-teen relationship is strengthened. When parents gained new knowledge about attachment, adolescent development, and parenting, it served as the foundation for increases in parental sense of self-efficacy, the capacity for emotional attunement with their child, and this supported the co-regulation process. This process promoted mutuality and partnership in the parent-teen relationship (see Figure 1 for thematic network analysis). While all FGDs informed the results, here we present illustrative quotes that most explicitly highlight the themes.

### Knowledge served as the foundation for change

The first organizing theme captured how parents learned about attachment, adolescent development, and parenting throughout the *eConnect* program. Parents described how the knowledge they gained from the program served as the foundation for change for themselves and within their relationship with their teens. For instance, a father remarked: “I got new knowledge; I came to know something I did not know before that opened a new door for me. I changed my behavior and the behavior of my children” (Somali FGD #2). Importantly, parents discussed how this new knowledge was especially valuable to them as newcomers. Parents expressed that learning about the norms, values, and expectations of parenthood in Sweden was fundamental to initiating relational change.

Parents also described how the new knowledge allowed them to re-evaluate their parenting practices and consider how this knowledge could be applied to their own parenting, leading them to adjust some of their rules, expectations, and other parenting practices. For instance, parents discussed gaining knowledge of the importance of peer relationships during adolescence and learning about teenager attachment needs related to peer relationships (e.g., peer belonging). Parents discussed how this knowledge informed shifts in their parenting practices: As one mother said: “I did not allow my children to hang with a friend or go outside with their friends, but now when they ask me if they can hang with friends, I allow and give them an agreed time, […] understanding that they need friends and play, and are going through the childhood stages” (Somali FGD #2). In another example, a mother described how she applied her knowledge of attachment needs (i.e., need for affection) to her behaviour towards to her children: “I benefited a lot from the course, such as telling your children how much you love them, so I repeatedly use that term [I love you] so that it gives happiness to the child” (Dari FGD #4).

### Increased parent self-efficacy

The second organizing theme described the parents’ increased belief in their ability to be successful in the parenting role after participating in the *eConnect* program. Many parents described that prior to the program, they felt stressed, overwhelmed, and lost in the parenting role. For instance, one father reported that from a cultural perspective, he was “taught to have children, but not how to raise them” (Somali FDG #1). Many parents described that after participation in the program, their confidence in the parenting role increased and felt more manageable. For example, one father shared that participation in the *eConnect* program “reduced the stress” that he previously experienced in the parenting role (Dari FDG #4).

Parents described this newfound sense of confidence particularly in navigating conflicts with their teen and in managing problems with their teens’ behaviours and emotions. This confidence was reflected in remarks made by parents: “I learned how to deal with my children if they come home being angry” (Arabic FGD #3) and “my knowledge has increased, for example, solving the behaviours coming with the child and childhood” (Somali FGD #2).

Many parents also discussed how their perceptions of themselves in the parenting role shifted as a result of participation in the program. Several parents mentioned the challenges of navigating the parenting role in a new context. For instance, one Arabic mother expressed: “I began to understand my parenting and developed in the parenting role.” (Arabic FGD #3). In another instance, another mother said: “before, I thought that I am not a good mother, but after the course, I learned that every problem has a solution and that my motherhood is not bad” (Somali FGD #1).

### Improved emotional attunement facilitates dyadic affect regulation

The third organizing theme described parents’ perception of becoming attuned to their child’s emotional experience, which supported the dyadic affect regulation process. Here, we define dyadic affect regulation as “the ability of a parent to step in and modulate the affective exchange between themselves and their teen, and to regulate their own emotional experiences and provide support when their teen feels overwhelmed” (Moretti et al., 2018, p. 380).

Parents described their efforts and ability to tune into their teen’s feelings. Many parents described learning to take a “step back” from their own emotional reactions, and to be curious about the emotions that were underneath their teen’s behaviours. For instance, one father described: “I learned to understand and follow up the conditions of my children’s mood, anger, happiness” (Somali FDG #2). Another Somali father remarked: “before the course, the children used to hide their needs and interests, but now I began to ask them their needs and feelings, how was your situation this week? How was your day?” (Somali FGD #1).

Many parents described instances where reflecting on their teen’s emotions enabled the regulation of their own emotions. Parents described how this reflection allowed them to respond to disruptive or upsetting situations with sensitivity and empathy. Parents repeatedly described the importance of responding in ways that were “patient,” “kind,” and “calm.” For instance, one mother noted: “when it comes to the children, I behave calmly, show patience and speak with them kindly, pray for God to protect them” (Somali FGD #2). Parents identified that these sensitive, attuned, and regulated responses facilitated the emotional regulation of their teens. As one mother shared: “before the course, when my child comes in home shouting and angry, I used to shout at them back, but after this course, I learned to know what makes the child angry and feel their problem that makes them shout” (Somali FGD #2). Another mother said: “when one of them [children] comes home and kicks the door, I speak calmly and say to him, are you angry today, calm down and take a shower, then he calms himself down.” (Somali FDG #2).

### Shifted power dynamics and emerging mutual parent-teen partnership

The final organizing theme relates to parents’ perceptions of the shift in power dynamics within the parent-child relationship. In parents’ descriptions of their relationship with their teens after participating in the program, a sense of mutuality and partnership was present. Many parents shared that in their home countries, parents held all the control and power within the parent-child relationships. Some parents reported they used to have strict expectations of child adherence to parental authority. Parents discussed how these expectations and relational ideas shifted over the course of the program. For instance, one father reported: “in Afghanistan and Iran, parents have the power and are always right […] parents force children to accept and listen to them regardless of whether it is right or wrong. Because parents are adults, children must obey what they say and think. But that is not the case here. We must listen and be more attentive to the children’s needs.” (Dari FDG #4). In another example, a mother remarked: “my method was that the child does things according to my instructions, reads their lessons, and behaves like an adult like me. When I participated in this course, I put to an end the restrictive style.” (Somali FGD #2).

Through participation in the program, parents began to view this power dynamic as unhelpful to their child and harmful to the parent-child relationship. For example, one mother described she learned: “how you can have a better dialogue, that threatening and intimidating them can’t —it doesn’t bring any result” (Arabic FGD #3). In another example, a father shared: “I used to get very angry sometimes, but now I have learned it is not good for my son” (Arabic FGD #3).

Parents’ descriptions of their relationships after participation in the program reflected an emerging sense of mutuality and partnership. Parents discussed their efforts to shift from a controlling way of parenting to seeking to listen, guide, and support their teens. One mother remarked: “my children knew the change, they asked me ‘mom, before you used to give us more orders, and you were very restrictive, what made you change?'” (Somali FGD #2). Parents described taking on a collaborative stance: Parents described their efforts to create a shared understanding of their own needs and interests and the needs and interests of their teens during discussions, and to work with their teens towards solutions. Parents also described being “open” in their communication with their teens. One father shared: “I stopped putting pressure on them, instead, we listen to each other and discuss frankly, I realized that we could agree easily when I listen to them, exchange ideas, and understood each other” (Somali FDG #2). In another example, a mother remarked: “he discusses with me whatever he wants like going outside, his friends, ‘mom I want to go that time' and everything. I say to them I do not reject you to go outside, but tell me the time you are going out, where you are going to, to call me if you are a bit late from our agreed time, so that I do not worry about you” (Somali FDG #2).

## Discussion

Our study explored the experiences of forcibly displaced parents from Syria, Somalia, and Afghanistan who participated in the *eConnect* program using post-program FGDs. Specifically, the study explored parent experiences of the program impact on the parent-teen relationship. Overall, parents expressed that participation in the *eConnect* program strengthened their relationship with their teenagers. This was related to enhanced knowledge about attachment, adolescent development, and the parenting role in a new context, which served as the foundation for increased self-efficacy in parenting, developing capacity for emotional attunement and dyadic affect regulation. Ultimately, this promoted greater mutuality and partnership in the parent-teen relationship. Previous quantitative research has shown that both the *Connect* and the *eConnect* program significantly increases parent-child attachment security (Bao & Moretti, 2023; Barone et al., 2020; Moretti et al., 2012; Moretti & Obsuth, 2009). Although these previous studies are quantitative in nature, our findings appear congruent with such research. Moreover, in our qualitative study, the parents elaborated on the shifted relational components that contributed to the meaningful improvements they experienced in their relationships with their teen.

While quality parent-child relationships are a key determining factor of healthy child development generally, quality parent-child relationships are especially critical in the context of trauma and adversity (McLaughlin & Lambert, 2017). For forcibly displaced families, both pre-displacement trauma and post-displacement acculturative stress place forcibly displaced youth at a high risk of mental health problems (Blakemore et al., 2020). The experience of forced displacement also gives rise to unique challenges in the parent-child relationship (e.g., acculturative dissonance). Our findings suggest that *eConnect* program may be a promising intervention to promote strengthened parent-teen relationships among forcibly displaced families. In fact, emerging research indicates that programs targeting parent-child relationships are most effective in supporting positive outcomes for forcibly displaced families (e.g., mental health outcomes, family cohesion; Gillespie et al., 2022; Minhas & Benipal, 2018).

Our findings suggest that there are several program components and processes that underlie the impact of *eConnect* on parent-teen relationships. First, parents expressed that their knowledge of attachment, adolescent development, and parenting practices improved throughout the *eConnect* program and served as the foundation for changes within the parent-teen relationship. The psychoeducational components of *eConnect* may be particularly important for forcibly displaced families. In the immigrant context, previous literature has underscored that once parents identify differences between their own cultural orientations and parenting styles and those of the host country, they start to reassess and adjust their parenting practices to better fit with the new social contexts faced by their family (Ochocka & Janzen, 2008). While parents may adjust their parenting practices in new social contexts, this does not mean that they discard traditional values and beliefs. Instead, traditional values and beliefs may be retained but the manner in which they are transmitted to their children may change. *eConnect* may provide newcomers an opportunity to reflect upon their own cultural parenting orientations and to develop and modify their parentings skills, reducing parent-child conflict (e.g., acculturation dissonance) which is often present when adapting to a new host country.

Through participation in the *eConnect* program, parents’ experienced increased sense of parental self-efficacy (i.e., belief in the ability to perform the parenting role successfully; Jones & Prinz, 2005), which contributed to strengthened parent-child relationships. Previous research has shown that high parental self-efficacy is linked with more sensitive and responsive parenting (Albanese et al., 2019). This finding also aligns with previous quantitative evaluations of the *Connect* program that have demonstrated significant increases in parenting self-efficacy after the program both in Canadian samples (Bao & Moretti, 2023; Moretti & Obsuth, 2009) and with forcibly displaced parents (Osman et al., 2017, 2021).

Parents’ discussions also reflected increased attunement to their teen’s emotions. Parents’ described learning to “step back” from their own emotional responses and “step into” their teen’s emotions. This emotional attunement facilitated the process of parent-teen dyadic affect regulation (i.e., parent ability to regulate their own emotions and support their teen in emotional interactions; Moretti et al., 2018). These findings reflect the established importance of dyadic affective attunement in building secure attachment relationships (Woltering et al., 2015). The findings are also consistent with our previous qualitative study that found that Somali-born parents became more aware of their teen’s emotions after participating in the *Connect* program (Osman et al., 2019), and other research that *Connect* significantly improves both parent and teen emotional regulation abilities (Benzi et al., 2023; Moretti et al., 2015).

Finally, our results suggest that after participation in the *eConnect* program, parents described shifted power dynamics and a sense of mutual partnership in the parent-teen relationship. Parents’ descriptions of their parenting practices before participation in *eConnect* program reflect an authoritarian parenting style (i.e., high levels of control and low levels of warmth, support, and responsiveness; highly demanding, punitive, and expectations of adherence to an absolute standard of behavior; Thompson & Baumrind, 2019). After the *eConnect* program, parents’ descriptions of their parenting reflect a more authoritative parenting style (i.e., high levels of warmth, support, and responsiveness; collaboration, open communication when limit setting, and promotion of child autonomy; Thompson & Baumrind, 2019). Importantly, an authoritative parenting style has been consistently associated with an array of positive developmental outcomes (Kuppens & Ceulemans, 2019), including secure parent-child attachment relationships (Doinita & Maria, 2015), compared to an authoritarian parenting style. Previous research has also identified that the shift from an authoritarian parenting style to a more authoritative parenting style is an important part of successful parenting transition for immigrants (Ochocka & Janzen, 2008; Osman et al., 2016). Our findings also reflect a previous *Connect* evaluation study that examined shifts in parental internal representations of the parent-child relationship before and after the program (Moretti et al., 2012). The study demonstrated that parent-child partnership and mutuality increased (e.g., open communication, reciprocity, partnership in responsibility), and that power struggles (e.g., conflicts) between parents and their teen reduced.

Taken together, these findings suggest that *eConnect* could be a promising intervention for supporting forcibly displaced parents, and strengthening parent-teen relationships. The study findings are especially notable, given that *eConnect* was implemented during the COVID-19 pandemic, a period where high levels of parental stress and familial conflict were reported (Dellafiore et al., 2022; Sinko et al., 2022). The COVID-19 pandemic prompted a shift to virtually delivered mental health care, and online interventions have shown promise in reducing barriers to service access beyond the COVID-19, including reaching those in geographically isolated or rural areas and creating possibilities for organizations to bring together facilitators speaking the native language of parents (Elisabeth et al., 2020). However, when delivering online interventions for forcibly displaced populations and parents from low socioeconomic groups, professionals must consider parents accessibility and literacy in technology (Darrat et al., 2021; Hynie et al., 2022).

### Limitations and Conclusions

This study is not without limits. While the FGDs were facilitated in parents’ native languages, the translation of the FGDs may have resulted in the loss of nuances in parents’ described experiences. However, the last author re-listened to the original audio recordings during the analytic process to ensure themes captured the parents’ experiences. Although the last author’s research expertise with forcibly displaced populations and the *Connect* program, and her lived experience as an immigrant, supports credibility of the study, this author may have had pre-conceived expectations that biased the findings. To mitigate this risk, the initial coding that formed the basis for analysis was also performed independently by the first author who does not share the lived experience or research expertise with this population. Future research could include the voices of the teens of parents participating in the program to explore if the relational changes described by the parents were also experienced by their teens. Despite limitations, the delivery and implementation of the *eConnect* program shows promise to support forcibly displaced families.

## Appendix

### Semi-Structured Interview Guide

1. Can you tell us about your experiences of participating in the parenting support program eConnect?
2. Can you describe more in detail the most valuable part of the parenting program eConnect?
3. What has been positive about participating in the eConnect program?
4. What has been negative about participating in the eConnect program?
5. Can you tell us what has been important about participating in the eConnect program? Why did you want to participate in the parenting program?
6. How have you used the knowledge learned from the parenting program with your family?
7. Can you tell us about the role plays and the examples? Were there examples you recognized or not recognized?
8. What made you accept this course?
9. Is there anything you missed in the course?
10. Are there any other aspects of the parenting program you would like to share with me?
